# Absence of correlation between ex vivo susceptibility to doxycycline and *pfteQ*–*pfmdt* gene polymorphism in French Guiana

**DOI:** 10.1186/s12936-015-0788-y

**Published:** 2015-07-25

**Authors:** Marie Mura, Sébastien Briolant, Damien Donato, Béatrice Volney, Stéphane Pelleau, Lise Musset, Eric Legrand

**Affiliations:** Laboratoire de Parasitologie, Centre National de Référence du Paludisme aux Antilles, Guyane, Institut Pasteur de la Guyane, Cayenne Cedex, France; Direction Interarmées du Service de Santé en Guyane, Quartier La Madeleine, BP 6019, 97306 Cayenne Cedex, French Guiana; Institut de Recherche Biomédicale des Armées, BP 73, 91223 Brétigny sur Orge Cedex, France; Unité de Recherche Génétique et Génomique des Insectes Vecteurs, Institut Pasteur, 25-28 rue du Dr Roux, 75724 Paris Cedex 15, France

**Keywords:** Malaria, Doxycycline, Molecular markers, Anti-malarial drug resistance, French Guiana

## Abstract

**Background:**

In French Guiana, doxycycline is used for both chemoprophylaxis and the treatment of malaria. The presence of isolates with reduced ex vivo susceptibility to doxycycline in French Guiana makes it critical to identify any genetic determinants contributing to the chemosusceptibility level of *Plasmodium falciparum* to doxycycline, such as *pfmdt* and *pftetQ*, which were recently identified as potential molecular markers in African isolates.

**Methods:**

A Bayesian statistical approach was used to define different ex vivo doxycycline phenotypes. The *pfmdt* and *pftetQ* gene copy numbers were quantified by quantitative real-time polymerase chain reaction in 129 *P. falciparum* isolates collected between 2000 and 2010, and *pftetQ*, *pfrps7*, *pfssurRNA*, and *pflsurRNA* sequences were analysed after amplification by polymerase chain reaction.

**Results:**

*PftetQ* and *pfmdt* copy numbers were not associated with reduced susceptibility to doxycycline in *P. falciparum* within French Guiana. Sequence analysis of the genes revealed five known single nucleotide polymorphisms. Three new SNPs were identified in the apicoplast ribosomal RNA long sub-unit (*pflsurRNA*): C740T, A1875C and A1875T. These polymorphisms were not associated with reduced chemosusceptibility to doxycycline.

**Conclusions:**

The present study does not validate *pfmdt* and *pftetQ* genes as molecular markers of decreased susceptibility to doxycycline in *P. falciparum* isolates in French Guiana.

## Background

Malaria remains a public health problem in French Guiana despite measures to strengthen health protection and disease prevention. It occurs in geographical foci located along the rivers and near gold-mining sites in the Amazonian forest [1], where the high number of illegal foreign workers unfortunately limits the impact of preventive measures. Self-medication using imported molecules with an unclear compliance contributes to the selection of drug-resistant parasites in the region [2]. *Plasmodium falciparum* accounts for 30% of the 1,000 malaria cases reported each year by the health regional agency [3]. As in the neighbouring countries of the Amazon basin, some strains of *P. falciparum* are resistant to chloroquine, amodiaquine, sulfadoxine–pyrimethamine, chloroquine–proguanil, halofantrine, and even quinine [4]. This justified the introduction of quinine–doxycycline combination therapy as a first-line treatment in 1995. It was replaced by the artemether–lumefantrine combination in 2002 but is still used to treat severe malaria in second-line treatment. Doxycycline is an antibiotic of the tetracycline family. Its anti-malarial activity has been known for 40 years following ex vivo [5, 6] and clinical studies [7–9]. The mode of action of this antibiotic on the *Plasmodium* parasite is not fully understood. In bacteria, cyclines inhibit protein synthesis by binding to protein S7 of the small ribosomal subunit and to various ribonucleic acids of the 16S ribosomal RNA, preventing the binding of aminoacyl-transfer RNA to site A of the ribosome and thus blocking the elongation step of translation [10]. In *P. falciparum*, the doxycyline mechanism of action targets two organelles, the mitochondria and the apicoplast. Cyclines also decrease the activity of an enzyme, dihydroorotate dehydrogenase, involved in the de novo synthesis of pyrimidines [11]. A related drug, minocycline, is also thought to decrease the transcription of mitochondrial genes (sub-unit I of cytochrome *c* oxidase and apocytochrome *b*) and plastid genes (sub-unit rpoB/C of RNA polymerase) [12]. Doxycycline appears to principally target the apicoplast in *P.falciparum*, and may block translation by binding to the small ribosomal sub-unit, causing a delayed death [13]. Doxycycline given on a daily basis has been shown to be an effective causal chemoprophylaxis [14]. It is now recommended by the French health authorities for chemoprophylaxis in countries with high prevalence of chloroquine resistance or multidrug resistance (group 3 countries), as in French Guiana [15]. French troops deployed in the illegal gold mines take 100 mg daily doses of doxycycline for prophylaxis. The ability of *P. falciparum* to rapidly develop resistance and the use of doxycycline for both chemoprophylaxis and the treatment of malaria in French Guiana impose a close monitoring of resistance to this drug. No clinical treatment failure has been reported so far, but doxycycline is always used in combination for treatment. Although reported cases of malaria under doxycycline chemoprophylaxis are mostly believed to have resulted from poor compliance [16], they could also be explained by resistance. It is critical to identify early signs of resistance before resistant strains become prevalent and compromise the clinical and prophylactic utility of the molecule. Indeed, Briolant et al. [17] identify an association between the *P. falciparum* metabolite drug transporter (*pfmdt*; PFE0825w) and *P. falciparum* GTPase TetQ (*pftetQ*; PFL171c) gene copy numbers, the *pftetQ* KYNNNN sequence polymorphism and a decreased ex vivo susceptibility to doxycycline in African *P. falciparum* isolates. The threshold of decreased susceptibility to doxycycline was established ex vivo at 35 µM [18].

This study first aimed to determine the distribution and range of 50% inhibitory concentrations (IC_50_) of doxycycline for 800 *P. falciparum* isolates assayed between 2000 and 2010 in French Guiana. In the second part, the association between the *pftetQ*, *pfmdt* copy numbers sequence polymorphisms of the *pftetQ*, *pfssurRNA*, *pflssurRNA*, *pfrps7* and with decreased susceptibility to doxycycline has been evaluated.

## Methods

### *Plasmodium falciparum* isolates

Between January 2000 and December 2010, 800 *P. falciparum* isolates were obtained from the different health centres of French Guiana and collected by the CNRCP (Centre National de Référence pour la Chimiosensibilité du Paludisme) hosted by the parasitology laboratory of the Institut Pasteur de la Guyane. Fifty per cent inhibitory concentration (IC_50_) to doxycycline was determined using the ex vivo isotopic method described by Le Bras et al. [19]. DNA was extracted from blood samples by QIAamp^®^DNA Blood (Qiagen) according to the manufacturer’s protocol.

### Distribution and range of IC_50_

The statistical analysis was designed to answer the specific question of whether *P. falciparum* has a different profile of susceptibility to doxycycline. Parasite susceptibility is expressed as the IC_50_. As a heterogeneous population is observed, the data are assumed to come from a univariate Gaussian mixture with k components. Each observation is assumed to come from one of the k components, and the label of the group from which each observation comes is unknown. The number of components, the means, variances and weights of the different components in the model are unknown, as well as the vector of allocations of the observations. The analysis was performed in two steps. First, reversible jump Monte Carlo Markov Chains (RJMCMC) samplers were used to choose a suitable number of k components. The RJMCMC sampler is described by Richardson et al. [20]. The only difference with the algorithm is that we implemented only birth and death moves, following Cappé et al.’s recommendations [21]. Once a relevant number of components had been chosen, standard Gibbs samplers were run to obtain estimates of the model parameters and classify the observations [22]. It is well known that these classical Markov Chain Monte Carlo techniques are not sufficient to cover all the parameter space; it can stay within a neighbourhood of a local mode and may fail to visit other important modes. In order to improve the exploration of the parameter space and thereby improve convergence, the RJMCMC and Gibbs samplers were embedded in a population-based algorithm. Because of the ‘label-switching’ problem caused by symmetry in the likelihood of the model parameters [23], the mixture components should be labelled before making inferences about the parameters. A classical ordering constraint was used, which is biologically relevant here. The algorithms were run for 50,000 burn-in iterations and 20,000 post-burn-in iterations. These numbers are classically sufficient to obtain reliable results. Moreover, each algorithm was run three times to check that results between two different runs were similar and that there was no convergence problem. This analysis was performed with R^®^ software (version 2.10.1).

### Quantification of the *pfmdt* and *pftetQ* gene copy numbers by TaqMan^®^ real-time PCR

TaqMan^®^ real-time polymerase chain reaction (PCR) was performed using ABI7300 (Applied Biosystems) to estimate the *pfmdt* (PFE08254w) and *pftetQ* (PFL1710c) gene copy numbers relative to the single-copy of *pfβtubulin* gene (PF10_0084) in 129 isolates from the different phenotypic subpopulations.

Primers and probes were used as describe by Briolant et al. [17]. Individual PCR were performed using 1X TaqMan Universal PCR Master Mix (Applied Biosystems), 6 µM forward and reverse primers, 4 µM probe, and 1 µL of template DNA in a final volume of 25 µL. The thermal cycling conditions were 50°C for 2 min, 95°C for 10 min, then 50 cycles of 95°C for 15 s and 60°C for 1 min for *pfmdt*, and 55°C for 10 s followed by 60°C for 1 min for *pftetQ*. Each sample was assayed in triplicate and analysed with SDS software (version 1.3; Applied Biosystems). The PCR efficiencies of all primers pairs were evaluated using a dilution series of *P. falciparum* 3D7 genomic DNA. The $2^{{ - \varDelta \varDelta {\text{C}}}}_{\text{T}}$ method of relative quantification, where C_T_ indicates the threshold cycle, was used to estimate the copy numbers of *pfmdt* and *pftetQ* genes with the formula $\varDelta \varDelta {\text{CT}} = \left( {{\text{C}}_{{{\text{T}},\;pfmdt}} {-}{\text{C}}_{{{\text{T}},\;pf\beta tubulin}} } \right)_{\text{sample}} {-}\left( {{\text{C}}_{{{\text{T}},\;pfmdt}} {-}{\text{C}}_{{{\text{T}},\;pf\beta ubulin}} } \right)_{\text{calibrator}}$ [24]. Genomic DNA extracted from *P. falciparum* 3D7, which has a single copy of each gene, was used for calibration, whereas *pfβtubulin* served as the control housekeeping gene in all experiments. Data were analysed using Epi info^®^ software (version 3.5.1). Differences in the *pfmdt* and *pftetQ* gene copy numbers between the phenotypic groups were tested using the Kruskal–Wallis test. Genotype proportions were compared using the Fisher exact test. Statistical significance was denoted by p ≤ 0.05.

### Gene sequence polymorphism analysis

Four genes, *pftetQ*, *pfssurRNA*, *pflsurRNA* and *pfrps7*, were amplified by PCR with oligonucleotide primer pairs designed using Primer 3^®^ software (version 0.4.0) (Table 1). As there was only a few isolates with an IC_50_ exceeding the threshold, it was initially chosen to sequence genes from only ten isolates with the highest IC_50_ [median 40.15 µM (min 29.82 µM; max 77.84 µM)] and ten isolates with low IC_50_ to doxycycline [median 4.83 µM (min 3.28; max 6.05)]. The reaction mixture consisted of 10× mix PCR Gold buffer^®^, 200 µM of dNTPs, variable concentration of MgCl_2_ (Table 1), 200 nM of forward and reverse primers, 4 UI/100 µL of Ampli Taq Gold DNA polymerase (Applied Biosystems) and 2 µL of DNA in a final volume of 50 µL. Thermal Mastercycler^®^ (Eppendorf) were programmed as follows: 94°C for 10 min then 35 cycles alternating 94°C for 30 s, hybridization temperature (Table 1) for 30 s and 72°C for extension at 1 min per 1,000 bp, followed by a 15-min final extension step at 72°C. The reaction products were sequenced by Millegen^®^ biotechnologies (Labège, France). Sequences were analysed using MEGA^®^ software (version 5.05). Epi Info^®^ software (version 3.5.1) was used to perform data analysis. Differences in DNA sequences (*pfssurRNA*, *pflsurRNA*, *pfrps7*, *pftetQ*) and in amino acid sequences [PfTetQ, PfRps7—itals—as before ? (No because is official nomenclature)] between the two groups were tested using the Kruskal–Wallis test.

**Table 1:** Forward and reverse primers, hybridization temperature and MgCL_2_ concentration used for polymerase chain reaction and sequence analysis

Gene	Forward Primer Reverse Primer	Tm; [MgCl_2_]
*PftetQ*	5′-ATGTTTAAAAGAGTTTACGTATATAAAAATTC 5′-GAAATGTTCATAAGAAATTGGTATATTATT	50°C; 3 mM
	5′-TTGTATGATAAATCCTAAACCAGATA 5′-TTCATCGTCCTTCTCACAAATTATAT	50°C; 3 mM
	5′-TCACGACAAATGTGCTAGATAC 5′-ATCATCATTTGTGGTGGATATATACAT	55°C; 3 mM
	5′-TTAAATATTTCAGATAACCTGGATAAAGA 5′-TTGGGATACACTTTATATATTAACACTTTT	55°C; 3 mM
*PfssurRNA*	5′-AAAGAATATCAAAGGCGAAAGC 5′-ACCCTTATCAAGAGTATGTTTTAACC	60°C; 4 mM
	5′-CTACTAGGGTATCTAATCCTATTTGCTA 5′-GATAGTAGTTCAATTCTACTTATTTCCAT	60°C; 2 mM
*PflsurRNA*	5′-TATAGACCCGAAATCAAATGATCTAATT 5′-TTTGGACCGTTATAGATACAGCCG	60°C; 3 mM
	5′-ACATCTGCCCAGTGCTATTATGTTA 5′-AGCTAATGGTGAGATTTGAACTCATAA	60°C; 3 mM
*Pfrps7*	5′-TTTCCATGATCTACATGCCCTA 5′-TTCTTTAGATTCAACTGGGGTTTT	60°C; 4 mM

## Results

### Distribution and range of IC_50_ in 800 isolates between 2000 and 2010

**Figure 1:**
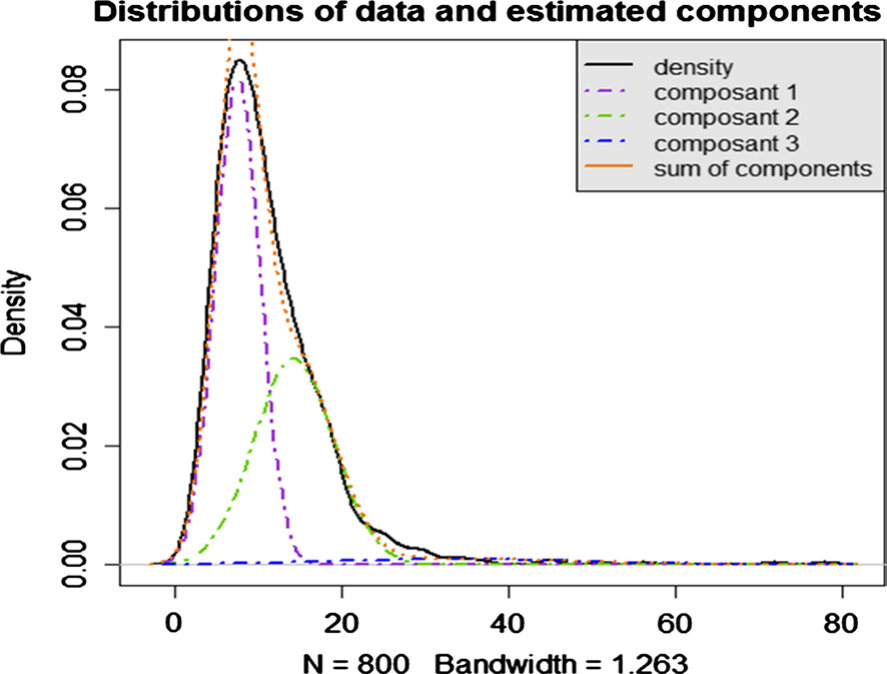
Distribution and density of the observed IC_50_, posterior components and sum of components.

Three runs of RJMCMC algorithms were performed, generating three chains of interest. All of these chains gave similar results for the number of components. Table 2 describes the mean values for the estimated posterior probabilities of the number of components in these three runs. The posterior distribution favoured four components, as the probability of k = 4 was the highest. Three runs of Gibbs sampler were performed and gave very similar results (Table 3). Figure 1 shows that the posterior Gaussian mixture fits quite well with the observed IC_50_ density.

**Table 2:** Estimated posterior probabilities of the number of components obtained on three runs

k = 1	k = 2	k = 3	k = 4	k = 5	k = 6	k = 7	k = 8	k = 9	k = 10
0.00	0.00	0.00	0.42	0.32	0.15	0.07	0.03	0.01	0.00

**Table 3:** Parameters of the Gaussian mixture with four components for the three runs

	run1	run2	run3	Mean
µ				
µ1	7.46	7.44	7.42	7.44
µ2	13.89	13.76	13.77	13.81
µ3	24.12	23.69	23.77	23.86
µ4	54.49	53.94	53.98	54.14
σ				
σ1	6.72	6.66	6.61	6.66
σ2	16.00	15.95	15.97	15.97
σ3	39.91	39.47	39.30	39.56
σ4	158.50	161.31	162.94	160.92
w				
w1	0.5439	0.5367	0.5358	0.5388
w2	0.3818	0.3816	0.3859	0.3831
w3	0.0633	0.0704	0.0672	0.0670
w4	0.0108	0.0112	0.0109	0.0110
n				
n1	481	477	480	479
n2	281	285	282	283
n3	32	32	32	32
n4	6	6	6	6
β	46.06	45.89	45.80	45.9

*µ* means, *σ* standard deviation, *w* weights of each components, *n* number of observation for each components, *β* β parameter.

### The *pfmdt* and *pftetQ* gene copy numbers

From 800 samples, 129 isolates were selected within the four phenotypic groups: 58 isolates [IC_50_ median 4.09 µM (min 0.7; max 11.1)] from group 1, 43 isolates [IC_50_ median 13.7 µM (min 11.2; max 21.96)] from group 2, 23 isolates [IC_50_ median 26.5 µM (min 22.2; max 37)] from group 3, and five isolates [IC_50_ median 50.92 µM (min 44.5; max 77.84)] from group 4. Only seven isolates had two or more copies of the *pftetQ* gene, showing no association with the phenotypic groups (Fisher test, p > 0.05). Among these isolates, five presented very low IC_50_ to doxycycline (group 1), and two presented high IC_50_ (group 3). Two copies of the *pfmdt* gene were found in only one group 3 isolate, with an IC_50_ of 35.8 µM. No statistical association was observed between the presence of multiple copies of *pfmdt* or *pftetQ* genes and a higher IC_50_ or belonging to phenotypic group 3 or 4.

### Gene sequence polymorphism

Sequence analysis of the four genes in ten isolates with high IC_50_ and 10 isolates with low IC_50_ revealed new single-nucleotide polymorphisms (SNPs) in the gene sequence of the long sub-unit ribosomal RNA (*pflsurRNA*), namely C740T (Genbank accession number: KM272854), A1875C (Genbank accession number: KM272855) and A1875T (Genbank accession number: KM272856). These SNPs were not previously noted in the PlasmoDB SNP database [25]. They were found in 35, 20 and 30% of isolates, respectively. Other SNPs were observed in the *pftetQ* and *pfssurRNA* genes, but neither they nor PfTetQ KYNNNN sequence polymorphism were significantly associated with the doxycycline IC_50_ phenotypes. No mutation was observed in *pfrps7*.

## Discussion

Doxycycline belongs to the tetracycline family of antibiotics. Tetracyclines are an effective anti-malarial, but their mechanism of action is much less clearly defined for *Plasmodium* than for bacteria. Ex vivo, doxycycline specifically impaired the expression of apicoplast genes and blocked apicoplast genome replication, leading to the loss of apicoplast function in the progeny of treated parasites and delayed death [13]. Most prophylactic failures of doxycycline against *P. falciparum* are associated with poor compliance [26] or inadequate low doses [27]. Doxycycline pharmacokinetic parameters could explain some of these cases, given its short elimination half-life (16 h) for slow action and delayed anti-malarial effect [28]. The early detection of reduced susceptibility or resistance to doxycycline entailed the monitoring of chemosusceptibility. A Bayesian mixture modelling approach was adopted to study doxycyline susceptibility phenotypes in the isolates circulating in French Guiana. All 800 IC_50_ values were classified into four components. All the isolates from group 4 and two isolates from group 3 had an IC_50_ value above the decreased susceptibility threshold of 35 µM [18]. This represents 1% of isolates. These data are consistent with clinical observations of limited prophylactic failures when doxycycline is used according to recommendations. Genotyping analysis was then necessary to try to identify the molecular basis of the susceptibility differences and correlate genetic profiles to the phenotypes.

Three potential molecular markers of decreased susceptibility were identified in African isolates, namely copy number variations of the *pfmdt* and *pftetQ* gene and PfTetQ KYNNNN sequence polymorphism [17]. These variations were also associated with decreased ex vivo susceptibility to doxycycline in isolates from the National Reference Centre for Imported Malaria in France mainland [29]. PfTetQ protein belongs to the translation elongation factor GTPase family, and possesses a plastid signal peptide [30]. It shares sequence similarity with several other bacterial resistance proteins [31]. Although the results showed find multiple copies of *pftetQ* gene in seven isolates, five of these had a very low IC_50_. It therefore follows that this genetic determinant is not associated with a decreased susceptibility to doxycycline in French Guiana and cannot be used to monitor doxycycline susceptibility. The protein product of the *pfmdt* gene corresponds to a putative metabolic drug transporter of the plasma membrane, and shows a similar sequence to the tetracycline resistance protein PfTetA, an efflux pump involved in bacterial resistance [32]. It was hypothesized that variations in the copy number of *pfmdt* gene can produce multiple efflux pumps and mediate resistance by increasing transport of doxycycline out of the cell. Previous research has demonstrated that an increased copy number of the *P. falciparum* multidrug resistant 1 (*pfmdr1*; PFE1150w) gene, which encodes an efflux pump of the vacuolar digestive membrane, is associated with mefloquine resistance in *P. falciparum* isolates from Southeast Asia [33]. Two copies of the *pfmdt* gene were found in just one isolate, with an IC_50_ of 35.8 µM. The other nine isolates which IC_50_ values corresponded to a decreased doxycycline susceptibility did not present multiple copies of *pfmdt*. This gene could therefore be involved in decreased doxycycline susceptibility but cannot be the only factor involved. Finally, PfTetQ KYNNNN sequence polymorphism was not associated with doxycycline IC_50_ phenotypes. A recent study on Kenyan isolates found a correlation between this polymorphism and a decreased susceptibility to doxycycline but not for *pfmdt* and *pftetQ* gene copy numbers. Surprisingly, multiple *pftetQ* gene copy numbers were associated with very low IC_50_ [34]. Further studies in other endemic regions are needed to test the reliability of these molecular polymorphisms as markers for decreased doxycyline susceptibility.

In this study, the results did shown observe any mutation in the *pfrps7* and *pfssurRNA* genes, although S7 and the small sub-unit ribosomal RNA are molecular targets of tetracyclines in bacteria [35, 36]. Three new SNPs were found in the DNA sequence of *pflsurRNA*, but without any association with a decreased susceptibility to doxycycline.

## Conclusions

Doxycycline is currently the only anti-malarial drug for which no *P. falciparum* treatment failure has been documented. The distribution of ex vivo susceptibility to doxycycline in French Guiana divided the population in four phenotypic groups. Only 1% of the 800 tested isolates had reduced susceptibility to doxycycline ex vivo, consistent with clinical observations. The present study does not validate the *pfmdt* and *pftetQ* gene as molecular markers of decreased susceptibility to doxycycline in *P. falciparum* French Guiana isolates.
